# Exploring the Genetic Signature of Body Size in Yucatan Miniature Pig

**DOI:** 10.1371/journal.pone.0121732

**Published:** 2015-04-17

**Authors:** Hyeongmin Kim, Ki Duk Song, Hyeon Jeong Kim, WonCheoul Park, Jaemin Kim, Taeheon Lee, Dong-Hyun Shin, Woori Kwak, Young-jun Kwon, Samsun Sung, Sunjin Moon, Kyung-Tai Lee, Namshin Kim, Joon Ki Hong, Kyung Yeon Eo, Kang Seok Seo, Girak Kim, Sungmoo Park, Cheol-Heui Yun, Hyunil Kim, Kimyung Choi, Jiho Kim, Woon Kyu Lee, Duk-Kyung Kim, Jae-Don Oh, Eui-Soo Kim, Seoae Cho, Hak-Kyo Lee, Tae-Hun Kim, Heebal Kim

**Affiliations:** 1 Department of Agricultural Biotechnology, Animal Biotechnology Major, and Research Institute for Agriculture and Life Sciences, Seoul National University, Seoul, 151–921, Republic of Korea; 2 Genomic Informatics Center, Hankyong National University, Anseong, 456–749, Republic of Korea; 3 CHO & KIM Genomics, Seoul National University Research Park, Seoul, 151–919, Republic of Korea; 4 Interdisciplinary Program in Bioinformatics, Seoul National University, Seoul, 151–742, Republic of Korea; 5 National Institute of Animal Science, RDA, Suwon, 441–706, Republic of Korea; 6 Korean Bioinformation Center, Korea Research Institute of Bioscience and Biotechnology, Daejeon, 305–806, Republic of Korea; 7 Swine Science Division, National Institute of Animal Science, RDA, Cheonan, 331–801, Republic of Korea; 8 Animal Research Division, Seoul Zoo, Seoul, 427–702, Republic of Korea; 9 Department of Animal Science and Technology, College of Life Science and Natural Resources, Sunchon National University, Suncheon, 540–950, Republic of Korea; 10 Optipharm, Inc., 63, Osongsangmyeong 6-ro, Osong-eup, Chengwon-gun, Chungcheongbuk-do, 363–954, Republic of Korea; 11 Laboratory of Developmental Genetics, College of Medicine, Inha University, Incheon, 400–103, Republic of Korea; 12 Department of Animal Sciencs, Iowa State University, Ames, Iowa, 50011, United States of America; University of Massachusetts, UNITED STATES

## Abstract

Since being domesticated about 10,000–12,000 years ago, domestic pigs (*Sus scrofa domesticus*) have been selected for traits of economic importance, in particular large body size. However, Yucatan miniature pigs have been selected for small body size to withstand high temperature environment and for laboratory use. This renders the Yucatan miniature pig a valuable model for understanding the evolution of body size. We investigate the genetic signature for selection of body size in the Yucatan miniature pig. Phylogenetic distance of Yucatan miniature pig was compared to other large swine breeds (Yorkshire, Landrace, Duroc and wild boar). By estimating the XP-EHH statistic using re-sequencing data derived from 70 pigs, we were able to unravel the signatures of selection of body size. We found that both selections at the level of organism, and at the cellular level have occurred. Selection at the higher levels include feed intake, regulation of body weight and increase in mass while selection at the molecular level includes cell cycle and cell proliferation. Positively selected genes probed by XP-EHH may provide insight into the docile character and innate immunity as well as body size of Yucatan miniature pig.

## Introduction

Pigs (*Sus scrofa domesticus*) were domesticated from Eurasian wild boars approximately 10,000–12000 years ago [1,2]. They have been domesticated in a wide variety of environmental conditions, and are currently found all over the world. Due to their tendency to grow and reproduce quickly, they are most often raised for human consumption and have become one of the main sources of protein for humans. To this end, swine breeds widely used in the livestock industry have been selected for traits of economic importance including average daily weight gain, longissimus muscle area, backfat thickness, and intramuscular fat content [3], and large body size. On average, swine breeds grow to be 4.8 kg at 3~4 weeks, 17.6 kg at 2.1~2.4 months, 53kg at 3.6~4.3 months, and 111kg at 6~8.3 months [4]. Duroc pigs known for its rapid growth rates, grow up to roughly 30kg by 10 weeks, 70kg by 16 weeks and 105kg by 22 weeks [5]. Similarly, Yorkshire pigs grow up to 29 kg by 10 weeks, 52.4 kg by 15 weeks and 82.6 kg by 20 weeks [6]. According to the Korea National Park Service, Korean wild boars are around 200kg when they are fully grown. It was reported that young male wild boar of 1.5 year age and 70 kg, female wild boars of 3~4 year age and 120~150 kg and male wild boars of 3~4 year age and 150~300 kg were observed at the town by local news journals in Korea.

Yucatan miniature pigs (YMP), however, grow slowly and even when fully grown are smaller than other pig breeds. The mean body weight of YMP is 31kg at 8 months and 83 kg at 24 months. (S1 Table). YMP have been bred from a naturally small breed, which was domesticated in the hot and arid Yucatan peninsula. It was imported into the United States in 1960 and since then, has been selected specifically for small body size for laboratory use and called YMP [7]. In 1970s and 1980s, they were studied at Colorado State University and were found to have distinct traits such as gentleness, intelligence, resistance to disease, and relative lack of odor [7,8,9]. For these characteristics, in addition to their size, YMP are used as laboratory animals worldwide.

Changes in the environment can act as a selective pressure and lead to the selection of organisms that are best suited for the new environmental conditions. In the case of domestic animals, selective pressure occurs as a combination of both natural selection (survival and reproductive ability in an enclosure) and artificial selection (intentional breeding by humans) [10]. These selective pressures have cherry-picked animals that have visibly higher fitness and led to the proliferation of these animals in the population. As the mutation that confer this higher fitness increases in frequency within the population, standing genetic variation at or near the mutation are either eliminated or reduced [11]. This process is known as a “selective sweep” and we can expect to find mutations affecting phenotypic traits by investigating regions of the genome with reduced variability or lack of standing variation. By comparing the YMP genome to other large swine breeds, signatures of selection specific to body size may be detected.

The aim of this study is to find genetic signatures of selective sweep in YMP using whole genome sequencing data of YMP, and other large pig breeds (Yorkshire, Landrace and Duroc). Whole genome sequencing data of 60 pigs were analyzed by the population statistic Cross Population Extended Haplotype Homozygosity (XP-EHH) to investigate the genetic characters of YMP, which derived from recent and artificial selection [12,13].

## Results

### Basic features about re-sequencing data of 70 pigs

In this study, 198,396,895 reads of average read counts were generated (from 148,854,532 reads to 335,518,129 reads) from re-sequencing of 70 pigs, which had 100 bp or 101 bp of planned read length, 406 bp of average distance between the paired reads (from 279 bp to 478 bp) and 44.2 bp of standard deviation of average distance (from 22.5 bp to 87.6 bp). Of total reads, 88 percent of reads were mapped to the reference genome (from 80.95% to 90.79%) (S2 Table). The reads covered 88 percent of genome (from 83.15% to 88.84%), and the sequencing data had average depth of 11.63 (from 4.93 to 15.08) nucleotides for the sequenced region and 10.26 (from 4.25 to 13.39) nucleotides for the whole genome region (S3 Table). For each swine sample, 6,625,510 SNPs were called averagely, and 29,368,693 SNPs were called in total (S2 and S4 Tables). SNP density of 10^6^-bp non-overlapping window for each swine breed is shown in S1 Fig.

**Fig 1:**
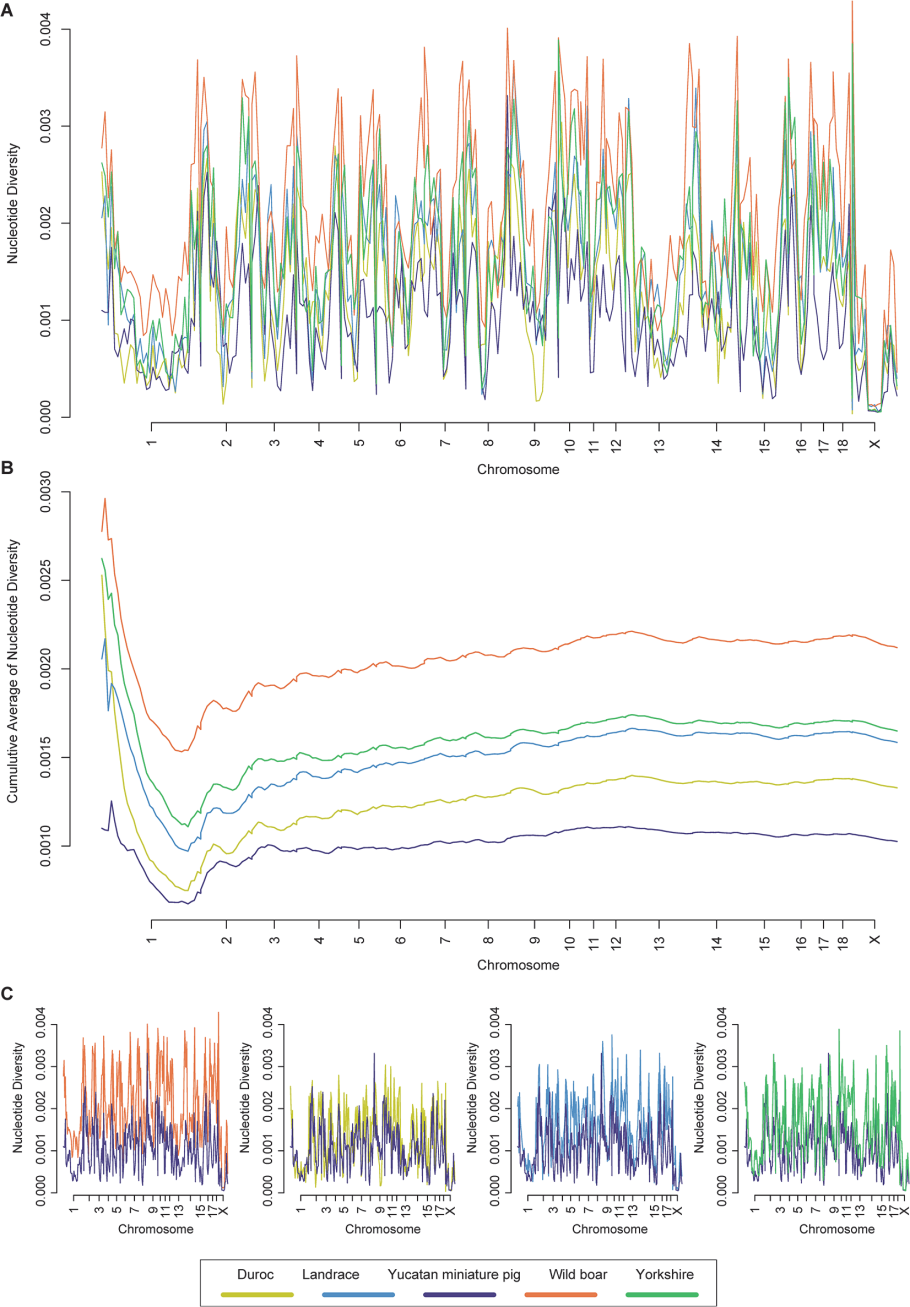
Nucleotide diversity (A and C) and cumulative average of nucleotide diversity (B) for each swine breed. Nucleotide diversity of 10^7^-bp non-overlapping window is shown. Each pig breed is marked by a colored line; orange for wild boar, blue for Landrace, green for Yorkshire, purple for Yucatan miniature pig and yellow for Duroc.

Nucleotide diversity of 10^7^-bp non-overlapping window was calculated, and *π* value and its cumulative average for each swine breed are shown in Fig 1A and 1B. Fig 1C shows pairwise *π* values between YMP and other swine breeds from Fig 1A. YMP had the lowest nucleotide diversity and Wild boar had the highest nucleotide diversity among 5 swine breeds.

### Phylogenetic tree, population admixture and principal component analyses

**Fig 2:**
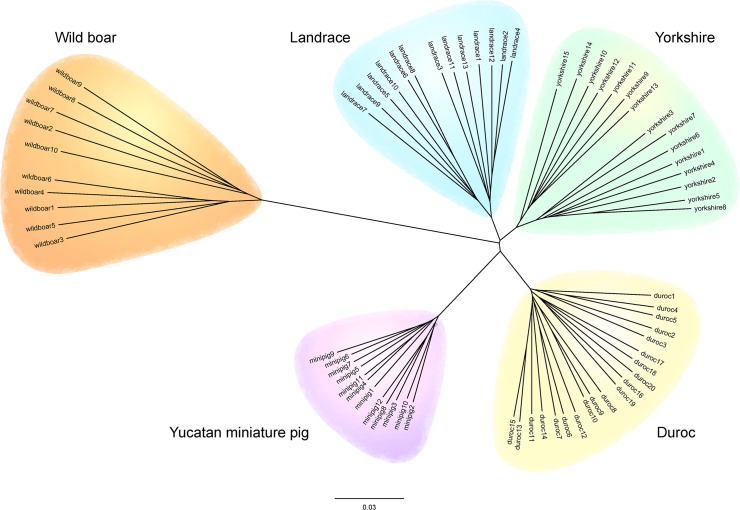
Unrooted phylogenetic tree of 70 pigs based on pairwise identity-by-state (IBS) distance from the entire autosomal SNPs. Each pig breed is marked by a colored circle; orange for wild boar, blue for Landrace, green for Yorkshire, purple for Yucatan miniature pig and yellow for Duroc. Scale bar indicate distance measure between each individual (1-IBS).

To assess the phylogenetic relationship among the pig breeds, we constructed an unrooted phylogenetic tree of 70 pigs based on pairwise identity-by-state (IBS) distance from the information of all autosomal SNPs. All individuals were grouped into five separate pig breeds as expected (Fig 2). Wild boars had the greatest genetic distance from the other breeds, followed by YMP with the next largest distance from Yorkshires, Landraces, and Durocs. Yorkshires and Landraces were genetically closer to each other than any other two breeds.

The estimated population admixture in the 70 analyzed pigs is shown in S2 Fig. We found that the five pig breeds were clearly distinguished when we assumed that the number of population (*K*) is 6. However, Yorkshire and Landrace shared components with each other.

Similar to the result of the unrooted phylogenetic tree, principal component analysis (PCA) scatter diagram showed clear division among the breeds with a similar pattern of distances between the groups (S3A and S4 Figs). Furthermore, Yorkshires and Landraces were not clearly distinguished in the PCA analyses whether it was conduct with all swine breeds or separately with the two breeds (S3B Fig).

### Signature detected by XP-EHH

The signatures of selection were identified based on a population comparison by the Cross Population Extended Haplotype Homozygosity (XP-EHH) test, which was devised to detect ongoing or nearly fixed selective sweeps by comparing haplotypes from two populations [14,15]. To get XP-EHH values, Extended Haplotype Homozygosity (EHH) and the log-ratio integrated EHH (iHH) were calculated using haplotype information of autosomal SNPs identified in the study. XP-EHH tests were performed in pairwise comparison between YMP and each of the large pig breed (Duroc, Landrace and Yorkshire). XP-EHH values are directional: extreme positive values imply selection in YMP while negative values suggest selection in other breed used in comparison. The distribution plot of raw XP-EHH values between YMP and other swine breeds is shown in S5 Fig. XP-EHH values have normal distribution.

For each comparison, we split the autosome into non-overlapping segments of 50 kb and the highest XP-EHH value was used as summary statistic for each window. Then the segments were grouped into 4 clusters according to their number of SNPs to take SNP frequency into account. For each cluster, empirical *p*-values of < 0.01 were used as cut off values to select significant regions in YMP. Under the assumption that the genes located within boundary of these regions are also outlier loci (genes), we selected genes associated with outlier loci (regions).

By XP-EHH test, we identified a total of 390 outlier regions and 429 associated genes in YMP from three comparative analyses: 164 regions (184 genes), 137 regions (146 genes), and 181 regions (196 genes) when compared to Duroc, Landrace, and Yorkshire, respectively (S7 Fig). There were 14 regions and 14 genes, which were detected by all three XP-EHH analyses. XP-EHH values, *p*-values and associated genes of outlier loci (regions) are shown in S5 Table.

**Fig 3:**
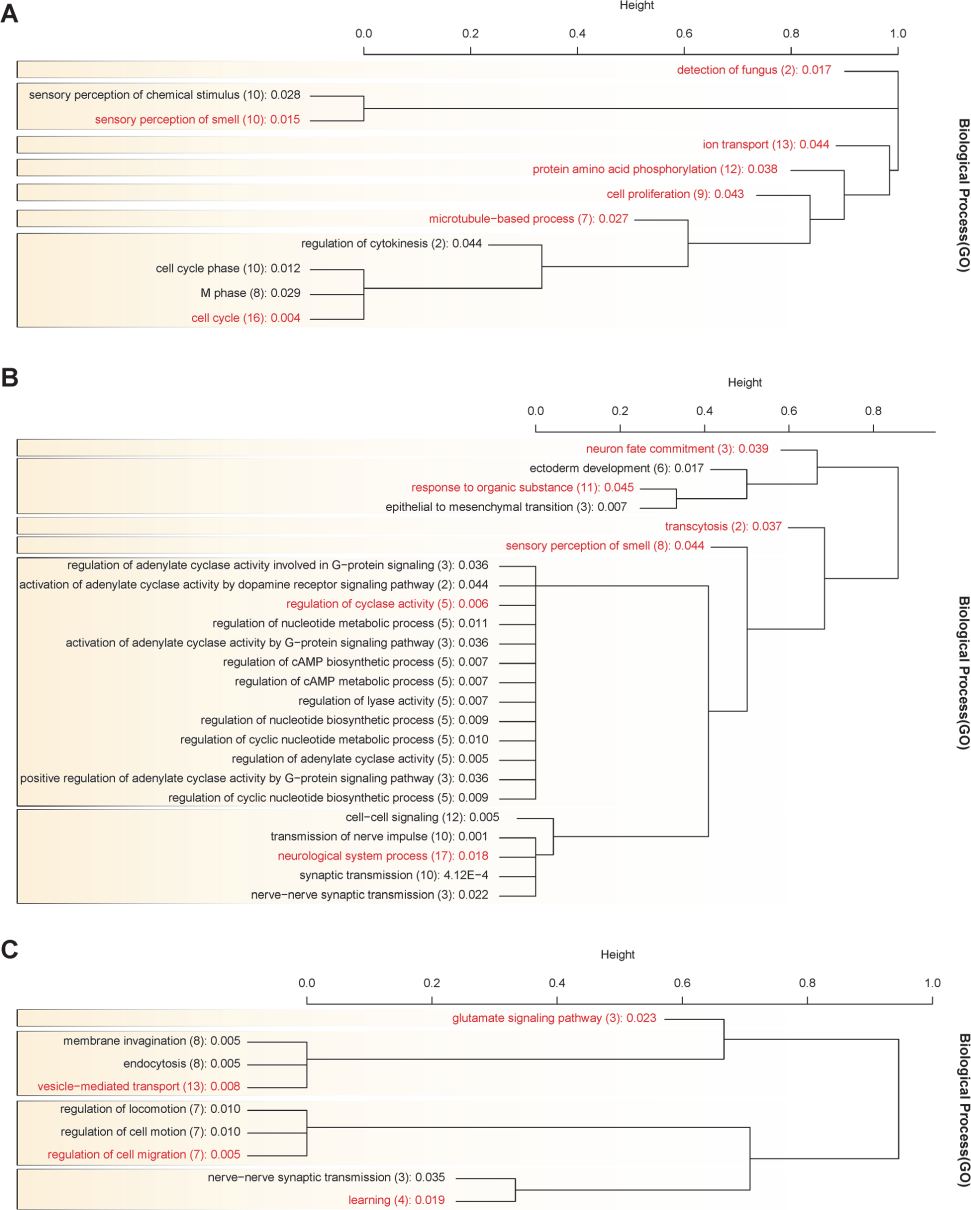
Hierarchical clustering of biological process GO terms associated with genes located in outlier loci (regions) detected by XP-EHH method in comparisons between Yucatan miniature pig and Duroc (A), Landrace (B) and Yorkshire (C). The gene list of each GO term clustered using DAVID was compared to calculate the distance between the GO terms. For a distance value of > 0.4~0.5, GO terms were re-clustered, and GO term groups are shown in boxes. The representative GO terms manually selected are shown in red. The numbers of genes in the GO terms are in brackets with the corresponding *p*-values.

For outlier loci (genes) in each comparison, we performed Gene Ontolgy (GO) term enrichment analyses with EASE < 0.05 to investigate their representation of functional groups (Fig 3). There were 45 enriched GO terms in all three comparisons. To reduce GO term list to the most relevant terms, we picked up representative GO terms based on the list of genes involved in. Representative GO terms associated with genes located in outlier loci (regions) detected by XP-EHH are shown in Table 1. The outlier loci (genes) between YMP and Duroc were mainly associated with detection of fungus, sensory perception of smell, ion transport, protein amino acid phosphorylation, cell proliferation, microtubule-based process and cell cycle in GO term. The outlier genes detected between YMP and Landrace were mainly associated with neuron fate commitment, response to organic substance, transcytosis, sensory perception of smell, regulation of cyclase activity and neurological system process. The outlier genes detected between YMP and Yorkshire were mainly associated with glutamate signaling pathway, vesicle-mediated transport, regulation of cell migration and learning. Nucleotide diversity plot of 14 genes in the intersection of all three XP-EHH analyses and 5 genes of interest which are known for their relation to growth is shown in S8 Fig.

**Table 1:** Representative biological process GO terms associated with genes located in outlier loci (regions) detected by XP-EHH method.

Comparison	Biological process GO term (No. of genes)
YMP vs Duroc	detection of fungus (2)
	sensory perception of smell (10)
	ion transport (13)
	protein amino acid phosphorylation (12)
	cell proliferation (9)
	microtubule-based process (7)
	cell cycle (16)
YMP vs Landrace	neuron fate commitment (3)
	response to organic substance (11)
	transcytosis (2)
	sensory perception of smell (8)
	regulation of cyclase activity (5)
	neurological system process (17)
YMP vs Yorkshire	glutamate signaling pathway (3)
	vesicle-mediated transport (13)
	regulation of cell migration (7)
	learning (4)

## Discussion

### Phylogenetic tree, population admixture and principal component analyses

Before investigating selection signatures, we first assessed the phylogeny of the pig breeds to evaluate the phylogenetic position of YMP within the species (Fig 2). An unrooted phylogenetic tree of 70 pigs identified clear division among the five pig breeds, as expected. Phylogenetically, YMP was distant from the wild boar group while being relatively close to Landrace, Yorkshire and Duroc groups. Wild boar had the greatest distance from the other breed groups. The distance between groups was concordant with previously reported phylogenetic trees, which were constructed by using neighbor-joining clustering and the unweighted pair group method with the arithmetic mean from microsatellite markers [16].

The distance pattern between the swine breeds was also identified by PCA (S3 and S4 Figs). However, Yorkshire and Landrace were not clearly distinguishable in either the PCA conducted with the five breeds or in a separate PCA of Yorkshire and Landrace, indicating the possibility of shared genetic components between the two breeds (S2 Fig). In the PCA of the two breeds, multiple eigenvectors accounted for similar proportion (5%) of the total variance in the genetic relationship matrix, limiting the power to stratify the two pig breed populations. This indicates that stratification using IBS is more sensitive than multiple eigenvectors.

### Signature detected by XP-EHH

For investigating recent selective sweep, we calculated XP-EHH values for YMP and other large swine breeds. XP-EHH tests were performed in pairwise comparison between YMP and each of the large pig breed that shows body size difference clearly (Duroc, Landrace and Yorkshire). XP-EHH statistic estimates haplotype differences between two populations and detects selective sweeps at or near fixation in one or the other population [13]. Thus, we expected being able to unravel the signatures of selection of body size in YMP.

The genes identified as being positively selected for in YMP are shown in S5 Table and the biological process GO terms associated these genes are shown in Fig 3. Representative GO terms from Fig 3 are shown in Table 1. According to GO term enrichment test results, the genes related to ‘cell proliferation’, ‘microtubule-based process’ and ‘cell cycle’ in comparison between YMP and Duroc (Fig 3A) could be involved in the growth and body size of YMP. Their function in regulation of cell cycle, cell division and cell proliferation are expected to affect the growth of YMP at the cellular level. The genes related to ‘response to organic substance’ and ‘transcytosis’ in comparison between YMP and Landrace (Fig 3B), and ‘vesicle-mediated transport’ and ‘regulation of cell migration’ in comparison between YMP and Yorkshire (Fig 3C) also could be involved in the growth and body size of YMP. Their roles involved in the process of change of cellular activity caused by stimulus of carbon containing molecule, transporting molecule across cell membrane and cellular component movement are important. They represent metabolism and increase or decrease of cellular component, and they are closely related to the regulation of cell cycle and cell proliferation, which could affect the growth of YMP at the cellular level.

We identified outlier loci (gene) *TGFB2* in comparison between YMP and Landrace, and between YMP and Yorkshire. The *TGFB2* gene (transforming growth factor, beta 2) encodes a member of the transforming growth factor beta (TGFB) family of cytokines, which has multiple functions, such as regulation of proliferation, differentiation, adhesion and migration [17]. TGF-betas are known to have critical roles in growth regulation and development, and have been studied extensively [18].

In Table 1 and Fig 3, the GO term, ‘sensory perception of smell’ was also enriched, suggesting a possible link between the senses and body size. Pigs are incredibly sensitive to smell, and adding garlic to feed as sweetener or modifying cereal inclusion rate has been shown to affect the palatability of meals which results in differences in the growth and meat producing performance [19,20].

The GO term ‘growth’ is defined as the increase in size or mass of an entire organism, a part of an organism or a cell. Therefore, genes detected by XP-EHH method and identified as being involved in growth term were thought to be related to the body size of YMP at the cellular level or at the level of organism. In all comparisons, we found 4 genes detected by XP-EHH and related to the ‘growth’ term, which are *APP*, *ACVR2B*, *PLEKHA1* and *CD 38* genes. *APP* (Amyloid Beta (A4) Precursor) gene encodes specific kind of cell surface receptor and transmembrane precursor protein, which are amyloid beta proteins and it is known as being genetically linked to familial Alzheimer’s disease. It was reported that *APP* knockout mice weighed 15%-20% less than wild-type mice [21]. *ACVR2B* (Activin Receptor Type-2B; Act RIIB) gene is a myostatin-related gene involved in the regulation and signaling of myostatin [22]. Transgenic mice expressing dominant-negative form of Act RIIB had increased muscle mass up to 125% more than those of control mice. Also, there was a study that haplotype structure at Act RIIB could explain the interindividual variation in skeletal muscle mass and strength in human [23]. *PLEKHA1* (Pleckstrin Homology Domain Containing, Family A (Phosphoinositide Binding Specific) Member 1) gene, also known as *TAPP1*, encodes a pleckstrin homology domain-containing adapter protein, which is localized to the plasma membrane of cell. This encoded protein has been studied for its role in regulating downstream of growth factor stimulation [24]. *CD38* gene is a multifunctional ectoenzyme widely expressed in cells and tissues. It was shown to be involved in regulating a wide variety of signaling pathways, such as those that regulate metabolism [25]. In the study of Barbosa et al. [26], *CD38* knockout mice had a higher metabolic rate compared to wild-type mice, and were protected against high-fat diet-induced obesity. The role of *CD38* was demonstrated as a regulator of body weight.

These genes involved in the biological process GO terms are not master gene such as *IGF1*, *BMP2* or *GH1* that directly affect body size as previously reported in mouse [27], dog [28], goat [29], pig [30] and human [31]. However, the genes identified in the study as having signatures of selection detected by XP-EHH method are related to the growth and body size. Thus, we found that genetic changes from the cellular level to the organismal level occurred in the evolutionary history of Yucatan Miniature Pig. However we could also find that the genes identified as being positively selected for in YMP are involved in neurological system and innate immunity.

Outlier loci (genes) (*PARK2*, *APP*, *CTNND2* and *DRD5*) were enriched for the GO term ‘learning’ in comparison between YMP and Yorkshire (Table 1 and Fig 3). In GO term, ‘learning’ means any process in an organism in which a relatively long-lasting adaptive behavioral change occurs as the result of experience. These involved genes are also related to the GO term ‘behavior’, which is defined as the specific actions or reactions of an organism in response to external or internal stimuli. In comparison between YMP and Landrace, the GO term ‘neurological system process’ was enriched, and *GRM5* (Glutamate Receptor, Metabotropic 5) gene was detected by all three XP-EHH analyses, which is one of the major genes of neurotransmission in central nervous system. This may affect intelligence or behavioral response to stimuli of YMP. Selection signatures in ‘learning’ and ‘neurological system process’, these indicate that the docile character of YMP might have evolved in the history of domestication.

Among 14 genes in the intersection of all three XP-EHH analyses, 5 genes were identified as being involved in innate immunity, which are *ITLN2*, *CLEC7A*, *OLR1*, *GABARAPL1* and *SH3BP2* (Table 2). *ITLN2* (Intelectin 2) gene has no known function yet. However, it has an important paralog *ITLN1*, which may play a role in the defense system recognizing bacterial cell wall [32], and *ITLN2* may play a role in the defense system by its sequence similarity. *CLEC7A* (C-Type Lectin Domain Family 7, Member A) gene encodes a member of C-type lectin/C-type lectin-like domain superfamily. It functions as a receptor that recognizes cell wall components of pathogenic bacteria and fungi, and many immune cells such as natural killer cells, macrophages and dendritic cells have this type of receptor and play important roles in the innate immune response [33]. *OLR1* (Oxidized Low Density Lipoprotein (Lectin-Like) Receptor 1) gene, also known as *LOX1* (lectin-type oxidized LDL receptor 1), also encodes a low density lipoprotein receptor that belongs to the C-type lectin superfamily, and it is thought to be involved in inflammatory response and antigen cross-presentation [34,35]. *GABARAPL1* (GABA(A) Receptor-Associated Protein Like 1) gene is involved in formation of autophagosomal vacuoles [36] and *SH3BP2* (SH3-Domain Binding Protein 2) gene has important function in regulating T-cell activation, natural killer cell activation and mast cell activation [37]. The result that these important immune related genes were found by all three XP-EHH analyses, indicates that innate immunity of YMP might have been changed in the evolutionary history.

**Table 2:** Outlier regions detected by all three XP-EHH tests between Yucatan miniature pig and each of large swine breed (Duroc, Landrace and Yorkshire) and associated genes.

Chromosome	Position start	Position end	Number of SNPs	XPEHH between YMP and Duroc	*p-*value of XPEHH between YMP and Duroc	XPEHH between YMP and Landrace	*p-*value of XPEHH between YMP and Landrace	XPEHH between YMP and Yorkshire	*p-*value of XPEHH between YMP and Yorkshire	Associated gene
4	97600000	97650000	790	3.73274	0.0039032	3.84921	0.0090749	4.28436	0.0050742	*ITLN2*
5	32200000	32250000	885	4.13872	0.0009758	3.85147	0.0086846	5.0409	0.0002927	*TBC1D30*
5	64550000	64600000	936	4.11254	0.0010734	4.53552	0.0013661	4.8002	0.0005855	*CLEC7A*,*OLR1*
5	64600000	64650000	148	2.56887	0.0082842	3.80914	0.0015907	3.19335	0.0081511	*GABARAPL1*
8	1150000	1200000	763	3.60337	0.0054645	4.26101	0.003025	4.39272	0.0031226	*SH3BP2*
8	5900000	5950000	754	3.61304	0.0053669	4.2187	0.0034153	4.05338	0.0083919	*ZNF518B*
9	24500000	24550000	985	4.05559	0.0014637	5.08135	9.76E-05	4.6132	0.0012685	*GRM5*
10	9850000	9900000	656	3.20103	0.0049394	3.94388	0.0036257	4.1702	0.0020004	*SPATA17*
10	9900000	9950000	1348	3.60608	0.0085831	4.67026	0.0037824	4.89801	0.002764	*SPATA17*
12	62550000	62600000	96	2.71247	0.0054344	3.80487	0.0016569	4.05116	0.0010603	*ALDH3A2*
13	20250000	20300000	793	3.82096	0.0029274	4.68363	0.0004879	4.2517	0.0056596	*CMTM6*
15	14900000	14950000	912	3.46727	0.0073185	3.95373	0.0066354	4.29819	0.0046838	*U6*
15	50000000	50050000	303	2.80518	0.004374	3.72619	0.0021209	3.55649	0.0035785	*7SK*
17	9650000	9700000	236	2.65267	0.0063623	4.60263	0.0001326	4.70793	0.0002651	*HGSNAT*

Thus, we could find the selection signatures involved in growth, body size, docile character and innate immunity of YMP using XP-EHH. Therefore, we suggest that the evolution in body size of YMP at the level of organ and organism, including feed intake, regulation of body weight and increase in mass of entire organism, and at the cellular level, including cell cycle and cell proliferation occurred during domestication process started about 10,000 years ago. We also suggest that the evolution related to docile character and innate immunity of YMP occurred simultaneously.

## Materials and Methods

### Sample preparation and whole genome re-sequencing

For the pigs experiment, the study protocol and standard operating procedures were reviewed and approved by the National Institute of Animal Science's Institutional Animal Care and Use Committee, Suwon, South Korea (approval No. 2009–077, C-grade, experiment for Landrace, Yorkshire and wild boar) and Seoul National University Institutional Animal Care and Use Committee, Seoul, South Korea (approval No. SNU-130527-8, experiment for Yucatan miniature pig,).

We used next generation sequencing data of 15 Yorkshires, 13 Landraces, 10 wild boars, 20 Durocs and 12 Yucatan miniature pigs to total 70 individuals.

For the different pig breeds, either blood or muscle samples were obtained for DNA extraction. Blood samples were collected from Landraces (seven male, six female) and Large Whites (seven male, eight female) from the National Institute of Animal Science, Korea. Blood (10 ml) was drawn from the carotid artery and was treated with heparin to prevent clotting. Muscle samples were collected from three male and seven female wild boars from the Southern part of Korea. For these samples, 3 μg of genomic DNA was randomly sheared using the Covaris System to generate ~300-bp inserts. The fragmented DNA was end-repaired using T4 DNA and Klenow polymerases, and Illumina paired-end adaptor oligonucleotides were ligated to the sticky ends. We analyzed the ligation mixture by electrophoresis on an agarose gel, and then purified the fragments. The purified DNA library was sequenced using the HiSeq2000 (Illumina, Inc). Clusters of PCR colonies were then sequenced on the HiSeq2000 platform using the manufacturer-recommended protocols.

Muscle samples from twenty male Durocs raised at a pig breeding company (Suncheon, Korea) were collected from a local slaughter house and YMP blood samples were collected from twelve females at a local laboratory pig distributor (Optipharm, Inc). Genomic DNA was extracted and a quality check was carried out using fluorescence-based quantification on an agarose gel, a standard electrophoresis on a 0.6% agarose gel and, via a pulsed-field gel, using 200 ng of DNA. Manufacturers’ instructions were followed to create a paired library of 500-bp fragments. This consisted of the following: purified genomic DNA fragments of less than 800 bp, fragments with blunt ends, fragments with 5′ phosphorylated ends, fragments with a 3′- dA overhang, some with adaptor-modified ends, purified ligation product, and a genomic DNA library. Following this, we generated sequence data using HiSeq2000.

Using the Burrows-Wheeler Aligner [38] with default settings, pair-end sequence reads were mapped to the reference pig genome (Sscrofa10.2). We used open-source software packages: Picard Tools, SAMtools [39], and the Genome analysis toolkit [40] for downstream processing and variant calling. Substitution calls were made with GATK UnifiedGenotyper [41] and all calls with a Phred-scaled quality of less than 20 were filtered out. For each chromosome, we simultaneously inferred the phased haplotype and imputed the missing alleles for the entire set of pig populations using BEAGLE [42]. Nucleotide diversity of 10^7^-bp non-overlapping window for each swine breed was calculated using PLINK [43].

### Estimation of phylogenetic tree and admixture in porcine population, and principal component analysis

Using GATK BeagleOutputToVCF [41], BEAGLE output files of each chromosome were converted to Variant Call Format (VCF) files. Then, the VCF files of autosome were converted to PLINK [43] input files using VCFtools [44]. From the information of all autosomal SNPs, population stratification was estimated using PLINK, which uses complete linkage agglomerative clustering based on pairwise identity-by-state (IBS) distance. In terms of IBS, there are three states between two individuals: 0, 1, or 2 shared alleles at a given locus. Symmetric matrix of IBS for all pairs of individuals was made, and these values ranged from 0 to 1, in which twin pairs had a value of 1. A distance matrix (1-IBS) for all pair of individuals was used to estimate a phylogenetic tree by neighbor-joining method [45]. Figures of the neighbor-joining trees were drawn using FigTree [46] (version 1.40).

Population admixture was investigated using STRUCTURE [47] (version 2.3.4), which carries out a model-based clustering method using genotype data. Due to limited computational power, we selected one out of every fifty SNPs to construct the STRUCTURE input file, and the population structure was estimated based on an admixture model.

Using the information of all autosomal SNPs, genetic relationship matrix (GRM) between pairs of individuals was estimated and principal component analysis (PCA) was performed by GCTA [48]. GCTA calculates eigenvectors, which are equivalent to those of EIGENSTRAT [49].

### Estimation of XP-EHH value and outlier loci detection

To detect selective sweep, Cross Population Extended Haplotype Homozygosity (XP-EHH) was calculated using the software xpehh [14] (http://hgdp.uchicago.edu/Software/).

For pairwise comparison of the Yucatan miniature pig and other large swine breed populations (Duroc, Landrace and Yorkshire), we used haplotype information of SNPs from the entire autosome to calculate Extended Haplotype Homozygosity (EHH) and the log-ratio integrated EHH (iHH). The distribution plot of raw XP-EHH values between YMP and other breeds is shown in S5 Fig. Then we split the autosome into non-overlapping segments of 50 kb and picked up the highest XP-EHH value as summary statistic for each window. The reason why we applied a maximum for XP-EHH to detect selection signature in YMP is that the XP-EHH values are directional: positive values imply selection in YMP while negative values suggest selection in other swine populations. To avoid bias towards windows have high SNP frequency in selecting significant windows, we grouped segments into 4 clusters according to their number of SNPs in increments of 350 SNPs (0 < cluster 1 < 350, 349 < cluster 2 < 700, 699 < cluster 3 < 1050 and 1049 < cluster 4). The number of SNPs in each window is shown in S6 Fig. Within each cluster, for each window *i*, the fraction of windows with a value of the statistic greater than that in *i* is defined as the empirical *p*-value, following the method previously reported [13,50,51]. We then chose the regions with empirical *p*-value < 0.01, which were considered to have strong signals in YMP. Genes related to these regions were determined by identifying genes located within boundary of regions.

In this study, empirical *p*-values were used as there are uncertainties in the demographic parameters making the use of *p*-values based on demographic models unreliable. A low empirical *p*-value for a locus implies that the locus is an outlier in comparison to the rest of the sampled loci in the genome. It has been reported that the use of empirical *p*-values has a tendency to under-represent the truly selected loci, especially when it comes to selection on standing variation and recessive loci [52].

### Gene ontology terms enrichment tests

The porcine Ensembl gene annotation (Ensembl Genes 78) and associated gene symbols were used for functional clustering and enrichment analyses using the Database for Annotation, Visualization and Integrated Discovery (DAVID) [53]. The representation of functional groups in Yucatan miniature pig relative to the whole genome was investigated using the Expression Analysis Systematic Explorer (EASE) tool [54] within DAVID. EASE is a modified Fisher’s exact test used to measure enrichment of gene ontology (GO) terms [55].

GO term enrichment tests were performed using EASE with a cut-off value of <0.05 for the outlier loci (genes) associated with XP-EHH.

### Ethics Statement

For the pigs experiment, the study protocol and standard operating procedures were reviewed and approved by the National Institute of Animal Science's Institutional Animal Care and Use Committee, Suwon, South Korea (approval No. 2009–077, C-grade, experiment for Landrace, Yorkshire and wild boar) and Seoul National University Institutional Animal Care and Use Committee, Seoul, South Korea (approval No. SNU-130527-8, experiment for Yucatan miniature pig,).

## Supporting Information

S1 Fig SNP density plot of 70 pigs. (TIFF)

S2 Fig Proportion of membership of 70 pigs into each of the five different breeds showing population admixture. (TIFF)

S3 Fig PCA scatter diagram showing the relative position of the five pig breeds (A) and the two closely related Yorkshire and Landrace breeds (B), defined by eigenvectors based on genetic relationship matrix from all autosomal SNPs. Eigenvector 1 and 2 accounted for 19 and 6% (A) and 5 and 5% (B) of the total variance in the genetic relationship matrix, respectively.
(TIFF)

S4 Fig 3-dimensional PCA scatter diagram showing the relative position of the five pig breeds defined by eigenvectors based on genetic relationship matrix from all autosomal SNPs. Eigenvector 1, 2 and 3 accounted for 19, 6 and 5.5% of the total variance in the genetic relationship matrix, respectively.
(TIFF)

S5 Fig Histogram showing raw XP-EHH score and frequency between Yucatan miniature pig and other swine breeds: Duroc (A), Landrace (B) and Yorkshire (C). (TIFF)

S6 Fig Histogram showing the number of SNPs in non-overlapping 50kb window and frequency. (TIFF)

S7 Fig Venn diagram showing the number of outlier loci (region) detected by XP-EHH in each comparison between Yucatan miniature pig and other breeds (Duroc, Landrace and Yorkshire). (TIFF)

S8 Fig Nucleotide diversity plot of 19 genes detected by XP-EHH. The nucleotide diversity was estimated for each 5-kb window. Each solid vertical line represents boundary of gene, and each dotted line represents boundary of region detected by XP-EHH. Each pig breed is marked by a colored line; blue for Landrace, green for Yorkshire, purple for Yucatan miniature pig and yellow for Duroc.
(TIFF)

S1 Table Body weight of Yucatan miniature pig. (DOCX)

S2 Table Mapping rate and the number of filtered SNPs of resequencing data of 70 pigs. (XLSX)

S3 Table Genome coverage and depth of resequencing data of 70 pigs. (XLSX)

S4 Table Number of SNPs in 70 pigs. (XLSX)

S5 Table Outlier regions detected by XP-EHH test between Yucatan miniature pig and each of large swine breeed (Duroc, Landrace and Yorkshire). (XLSX)
