# Supplementary figures and images for: Exploring the Genetic Signature of Body Size in Yucatan Miniature Pig
Source: PLoS One. 2015 Apr 17;10(4):e0121732. doi: 10.1371/journal.pone.0121732 (PMC4401510; doi:10.1371/journal.pone.0121732)

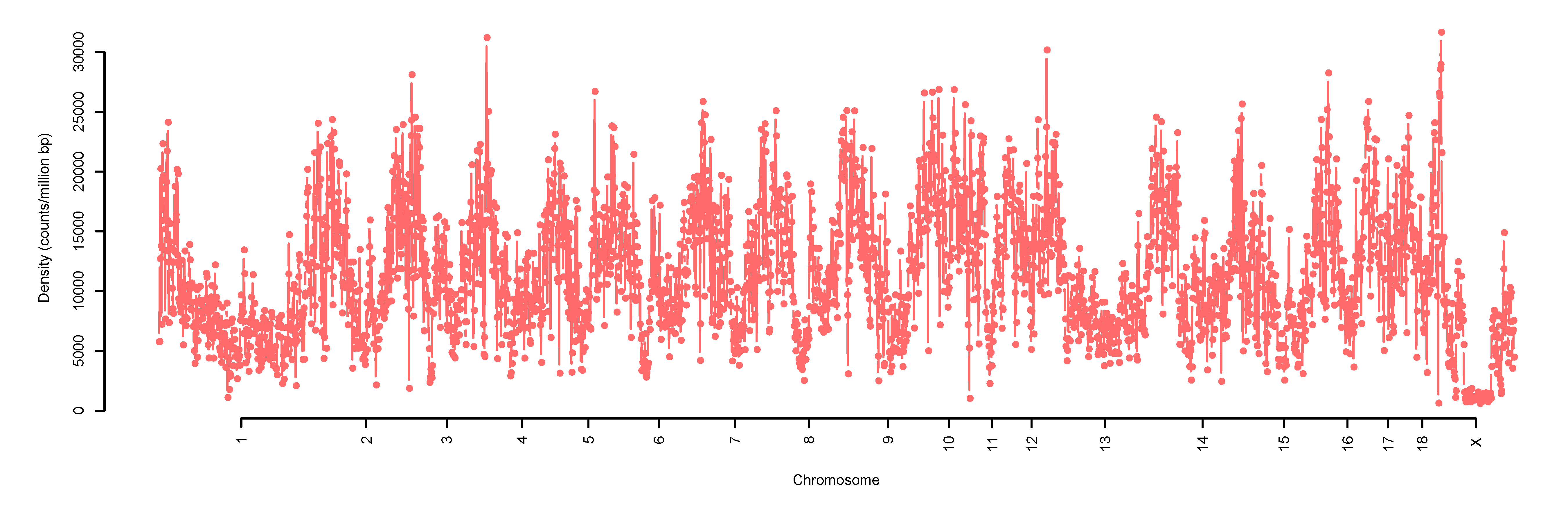

Supplement: S1 Fig — (TIFF) [file pone.0121732.s001.tiff]

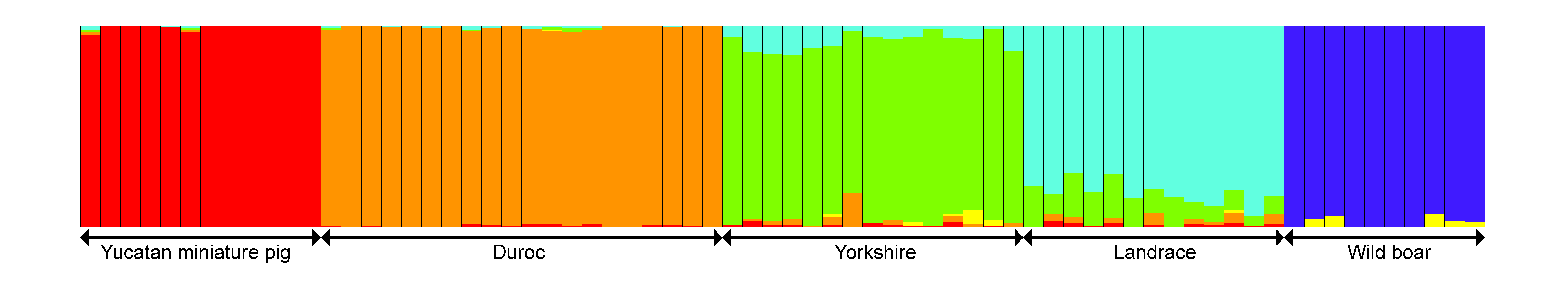

Supplement: S2 Fig — (TIFF) [file pone.0121732.s002.tiff]

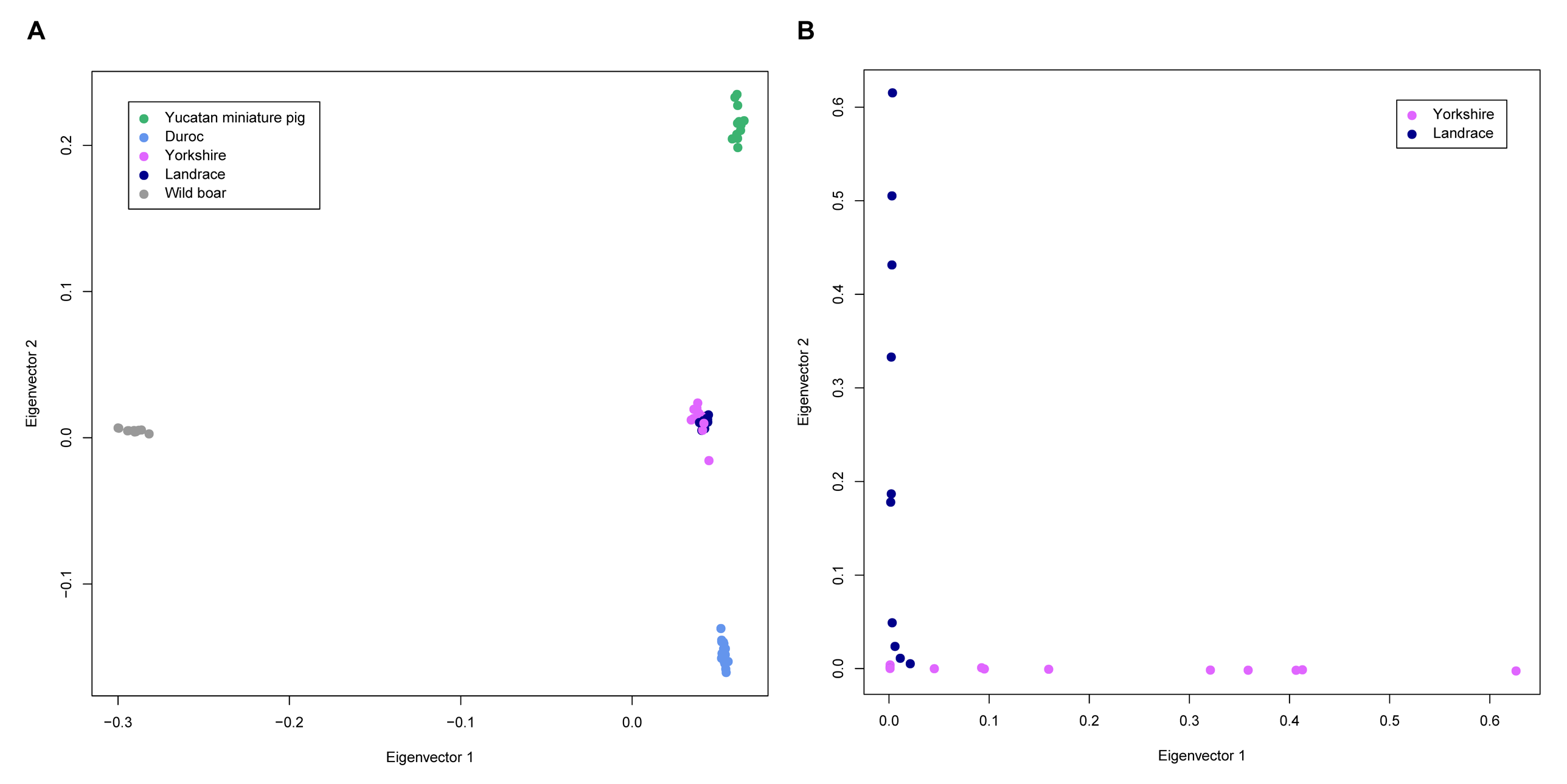

Supplement: S3 Fig — Eigenvector 1 and 2 accounted for 19 and 6% (A) and 5 and 5% (B) of the total variance in the genetic relationship matrix, respectively. (TIFF) [file pone.0121732.s003.tiff]

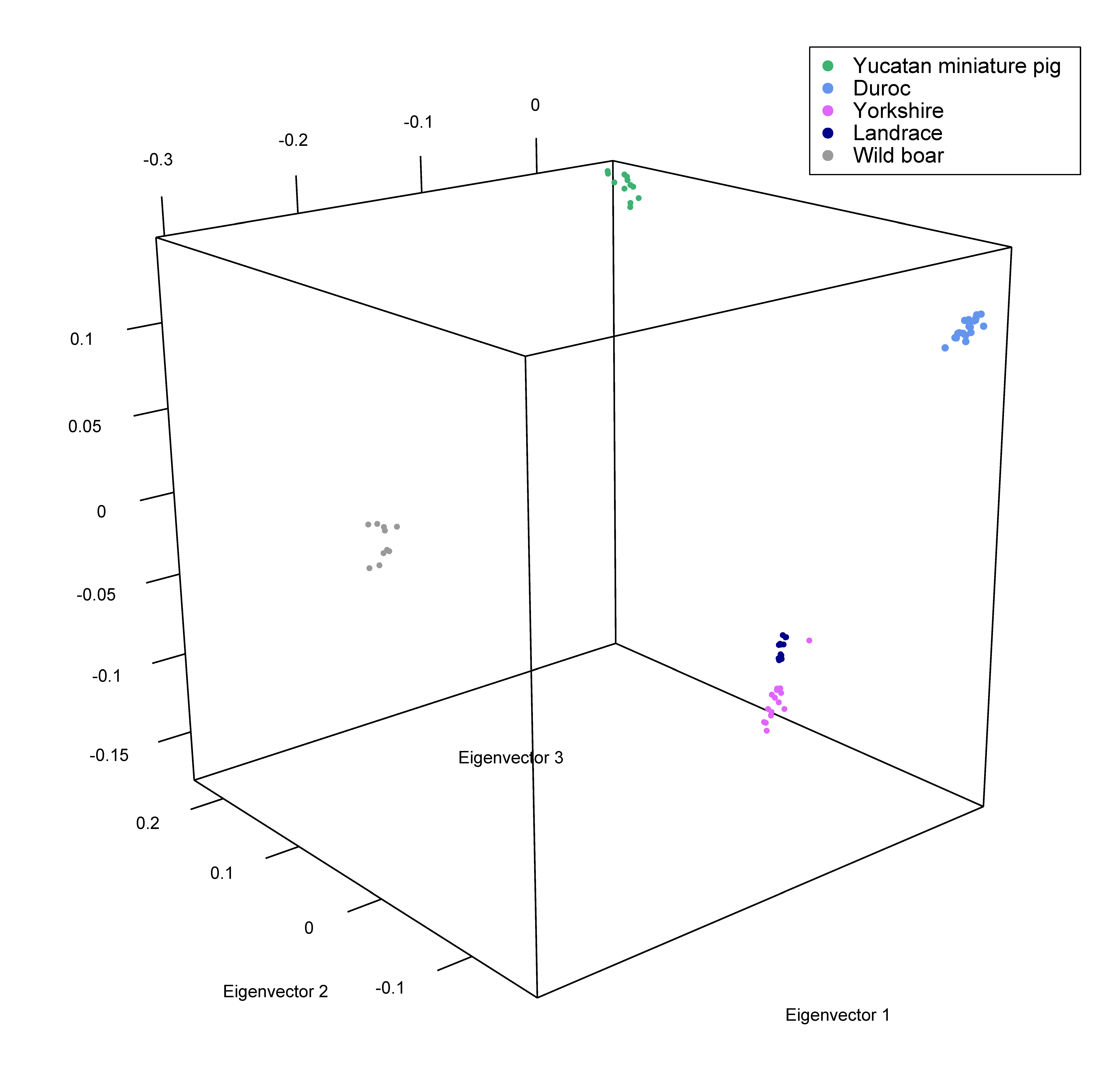

Supplement: S4 Fig — Eigenvector 1, 2 and 3 accounted for 19, 6 and 5.5% of the total variance in the genetic relationship matrix, respectively. (TIFF) [file pone.0121732.s004.tiff]

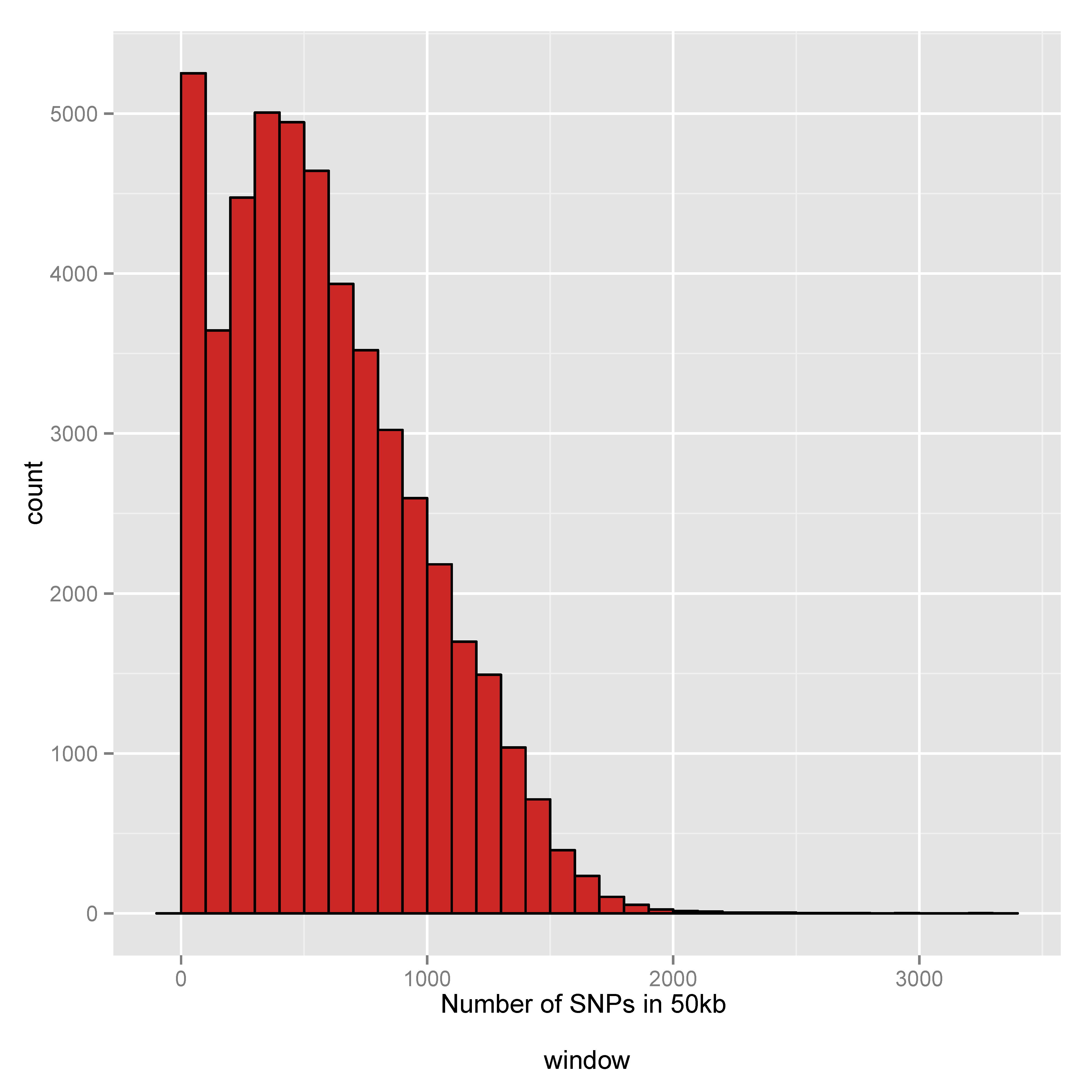

Supplement: S6 Fig — (TIFF) [file pone.0121732.s006.tiff]

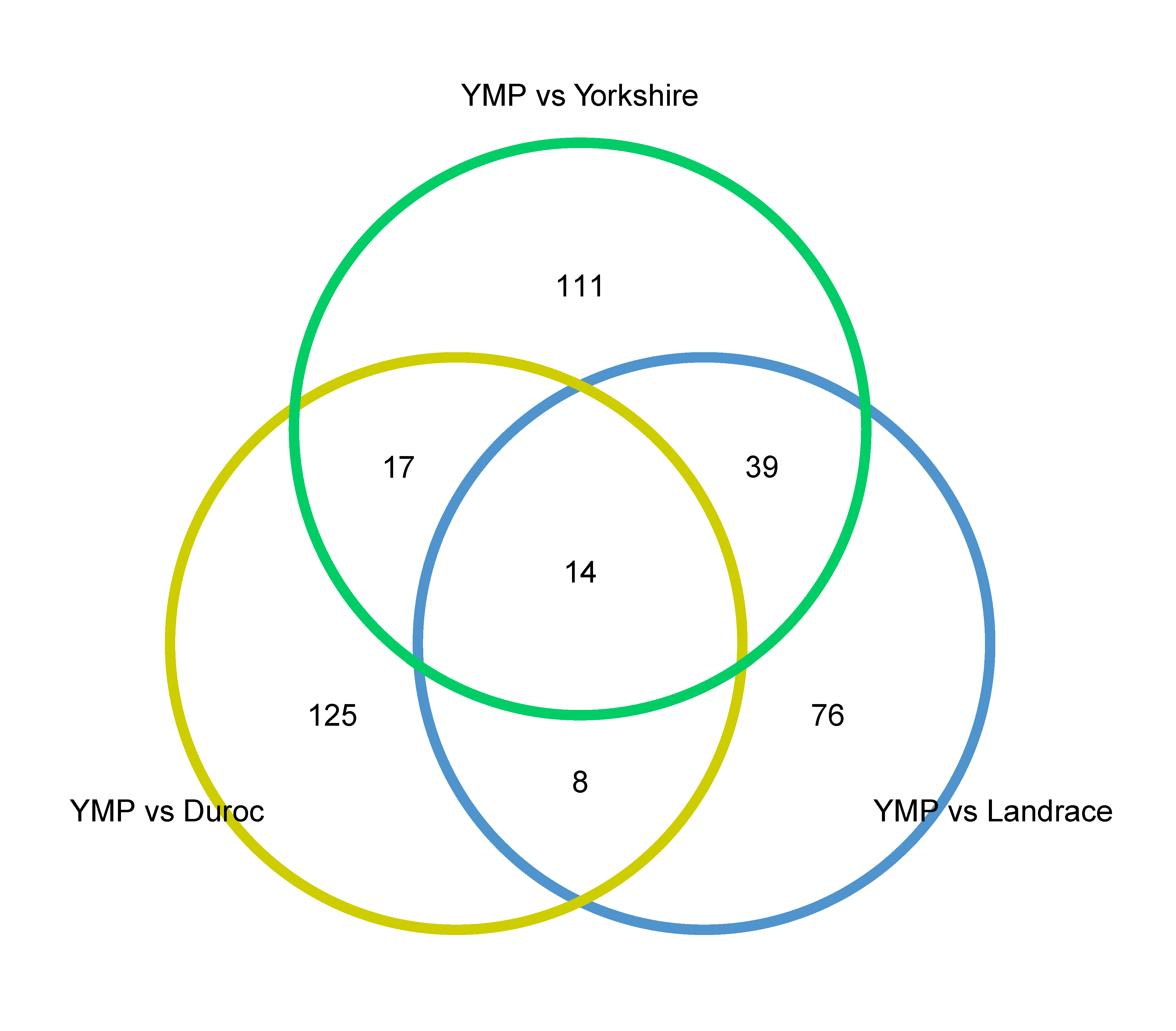

Supplement: S7 Fig — (TIFF) [file pone.0121732.s007.tiff]
