# Supplementary material for: Exploring the Genetic Signature of Body Size in Yucatan Miniature Pig
Source: PLoS One. 2015 Apr 17;10(4):e0121732. doi: 10.1371/journal.pone.0121732 (PMC4401510; doi:10.1371/journal.pone.0121732)
Supplement: S1 Table — (DOCX) [file pone.0121732.s009.docx]

**Table S1. Body weight of Yucatan miniature pig*****

| Age* | Female | | | Male | | |
| --- | --- | --- | --- | --- | --- | --- |
|  | Average** | Maximum** | Minimum** | Average** | Maximum** | Minimum** |
| 0 | 0.88 | 1.24 | 0.38 | 0.88 | 1.24 | 0.48 |
| 1 | 3.94 | 6.63 | 1.60 | 3.92 | 6.04 | 1.80 |
| 2 | 7.68 | 10.40 | 4.70 | 7.72 | 11.00 | 4.40 |
| 3 | 11.34 | 15.10 | 7.50 | 11.31 | 14.80 | 5.63 |
| 4 | 15.19 | 19.10 | 12.03 | 15.15 | 19.30 | 8.90 |
| 5 | 19.01 | 23.60 | 13.50 | 18.88 | 25.00 | 12.20 |
| 6 | 23.15 | 28.50 | 15.30 | 23.22 | 30.50 | 15.70 |
| 7 | 27.80 | 36.50 | 18.20 | 27.50 | 38.00 | 21.00 |
| 8 | 31.00 | 42.80 | 22.50 | 32.51 | 43.90 | 25.30 |
| 9 | 37.07 | 44.60 | 29.50 | 39.53 | 50.00 | 31.50 |
| 10 | 39.75 | 51.35 | 33.60 | 42.09 | 52.00 | 36.00 |
| 11 | 43.66 | 54.00 | 37.50 | 48.35 | 56.50 | 41.00 |
| 12 | 44.73 | 56.00 | 37.80 | 55.16 | 63.00 | 49.00 |
| 14 | 53.18 | 68.00 | 43.00 | 63.90 | 75.30 | 55.30 |
| 16 | 57.94 | 65.00 | 49.80 | 67.67 | 75.50 | 60.50 |
| 18 | 65.43 | 71.00 | 57.00 | 77.50 | 78.00 | 77.00 |
| 24 | 83.00 | 93.00 | 77.00 | 87.50 | 89.00 | 86.00 |

*Units are month.

**Units are kg.

***Body weight data was provided by Optipharm, Inc.
